# Pre-pandemic to early-pandemic changes in risk of household food insecurity among Maryland families with children

**DOI:** 10.1017/S136898002100481X

**Published:** 2021-12-10

**Authors:** Alysse J Kowalski, Ann Pulling Kuhn, Hannah G Lane, Angela CB Trude, Helina Selam, Erin R Hager, Maureen M Black

**Affiliations:** 1 Department of Pediatrics, University of Maryland School of Medicine, Baltimore, MD 21201, USA; 2 Department of Population Health Sciences, Duke University School of Medicine, Durham, NC, USA; 3 Department of Epidemiology and Public Health, University of Maryland School of Medicine, Baltimore, MD, USA; 4 RTI International, Research Triangle Park, NC, USA

**Keywords:** COVID-19, Pandemic, Food insecurity, CARES stimulus payment, School meals, Health disparities

## Abstract

**Objective:**

The objective was to examine risk and protective factors associated with pre- to early-pandemic changes in risk of household food insecurity (FI).

**Design:**

We re-enrolled families from two statewide studies (2017–2020) in an observational cohort (May–August 2020). Caregivers reported on risk of household FI, demographics, pandemic-related hardships, and participation in safety net programmes (e.g. Coronavirus Aid, Relief, and Economic Security (CARES) stimulus payment, school meals).

**Setting:**

Maryland, USA.

**Participants:**

Economically, geographically and racially/ethnically diverse families with preschool to adolescent-age children. Eligibility included reported receipt or expected receipt of the CARES stimulus payment or a pandemic-related economic hardship (*n* 496).

**Results:**

Prevalence of risk of FI was unchanged (pre-pandemic: 22 %, early-pandemic: 25 %, p = 0·27). Risk of early-pandemic FI was elevated for non-Hispanic Black (adjusted relative risk (aRR) = 2·1 (95 % CI 1·1, 4·0)) and Other families (aRR = 2·6 (1·3, 5·4)) and families earning ≤ 300 % federal poverty level. Among pre-pandemic food secure families, decreased income, job loss and reduced hours were associated with increased early-pandemic FI risk (aRR = 2·1 (1·2, 3·6) to 2·5 (1·5, 4·1)); CARES stimulus payment (aRR = 0·5 (0·3, 0·9)) and continued school meal participation (aRR = 0·2 (0·1, 0·9)) were associated with decreased risk. Among families at risk of FI pre-pandemic, safety net programme participation was not associated with early-pandemic FI risk.

**Conclusions:**

The CARES stimulus payment and continued school meal participation protected pre-pandemic food secure families from early-pandemic FI risk but did not protect families who were at risk of FI pre-pandemic. Mitigating pre-pandemic FI risk and providing stimulus payments and school meals may support children’s health and reduce disparities in response to pandemics.

Restrictions enacted in spring 2020 to slow the spread of the Coronavirus Disease 2019 (COVID-19) in the US disrupted daily life and led to widespread closures of businesses, schools and childcare centres. Estimates suggest food insecurity (FI) tripled among households with children, with over half of the increase attributable to the spike in unemployment^(1)^. COVID-19-related healthcare and caregiving expenses may have further contributed to financial hardship^(2)^. The pandemic exacerbated pre-existing disparities in FI, with low-income families and families of colour experiencing FI at higher rates^(3)^. In families with children, FI is associated with worse general health, behavioural problems, poor development and academic performance, and often co-occurs with excess weight gain^(4–8)^.

In response to the COVID-19 pandemic, federal safety net programmes were designed and expanded to alleviate pandemic-related economic hardships (Fig. 1)^(9,10)^. In March 2020, Congress passed the Coronavirus Aid, Relief, and Economic Security (CARES) Act which provided families with a one-time payment of up to $1200 per eligible adult and $500 per dependent child, expanded unemployment insurance eligibility, and provided a weekly supplement to state unemployment benefits from March to July 2020^(11)^. Eligibility for the CARES stimulus payment included having a Social Security number and IRS-determined 2019 adjusted gross income of < $75 000 for single and < $150 000 for married couples filing joint returns^(12)^. Several changes were made to United States Department of Agriculture (USDA) food assistance programmes to support food security, including modifications to the Supplemental Nutrition Assistance Program (SNAP) that allowed more families to receive the maximum monthly benefit and expanded online purchasing. In response to school closures, USDA implemented waivers for Child Nutrition Programs that reduced logistical and administrative barriers and allowed schools and community organisations to serve meals to children without cost to the families served^(13)^. Additionally, families eligible for free or reduced-price meals through the Community Eligibility Provision pre-pandemic were provided direct payments valued at the cost of each meal through the newly developed Pandemic Electronic Benefit Transfer (P-EBT) programme.

**Fig. 1:**
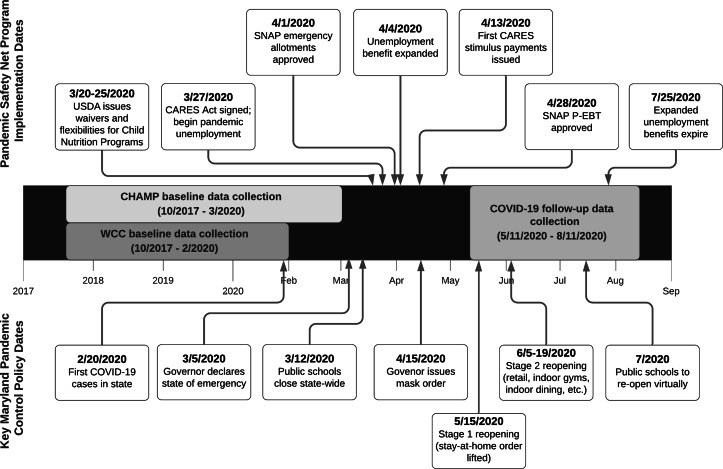
Data collection timeline (middle) with safety net programme implementation dates (top) and key state pandemic control policy dates (bottom) through August 2020. USDA, United States Department of Agriculture; CARES, Coronavirus Aid, Relief, and Economic Security; SNAP, Supplemental Nutrition Assistance Program; P-EBT, Pandemic Electronic Benefit Transfer; CHAMP, Creating Healthy Habits Among Maryland Preschoolers; WCC, Wellness Champions for Change

Changes in risk of household FI at the onset of the pandemic and the extent to which COVID-19 illness and economic hardships exacerbated FI risk and, conversely, safety net programmes alleviated FI risk are not well understood. Building on two ongoing statewide studies of families with preschool to adolescent-aged children that began prior to the pandemic, we conducted a rapid response survey from May to August 2020. We tested three hypotheses: (1) lower-SES households, households of colour and rural households were more likely to report early-pandemic FI risk, (2) COVID-19 illness and economic hardship increased the risk of early-pandemic FI and (3) safety net programmes protect families from FI risk.

## Methods

### Study population

We recruited participants from two ongoing statewide childhood obesity prevention intervention trials in childcare or school settings, Creating Healthy Habits Among Maryland Preschoolers (CHAMP) and Wellness Champions for Change (WCC)^(14,15)^. Both trials aimed to improve child diet and physical activity and enrolled three cohorts over three academic years (2017, 2018 and 2019).

**Fig. 2:**
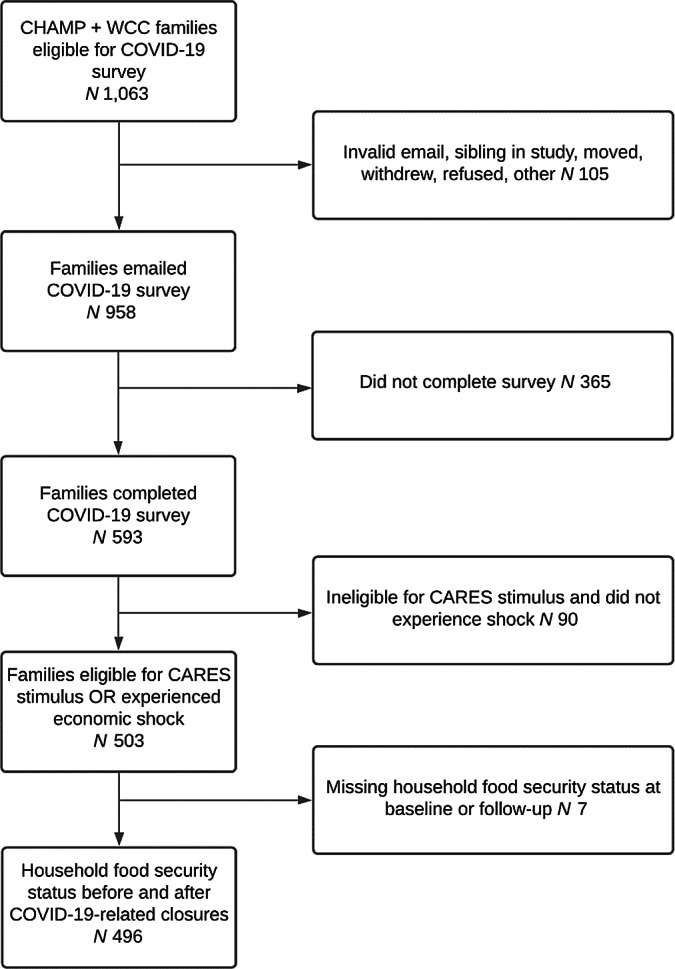
Analytical sample participant flow diagram. CHAMP, Creating Healthy Habits Among Maryland Preschoolers; WCC, Wellness Champions for Change; CARES, Coronavirus Aid, Relief, and Economic Security

The CHAMP and WCC studies took place in fifty-four childcare centres and thirty-three schools (eighteen elementary and fifteen middle) serving low- and middle-income communities in thirteen counties. Childcare centres were eligible if they accepted childcare vouchers, participated in the Child and Adult Care Food Program, or cost less than $300/week per child. Elementary and middle schools were eligible if > 40 % of the student body was eligible for free or reduced-price school meals. In spring 2020, 1063 caregivers who had completed a pre-pandemic baseline survey were invited to re-enroll in the COVID-19 study and 593 (56 %) re-enrolled through email, text or phone (Fig. 2).

### Data collection

We used data collected from caregiver surveys at two time points: pre-pandemic (10/2017 to 3/2020) and early-pandemic (5/11/2020 to 8/11/2020; 86 % completed in May–June) (Fig. 1). The pre-pandemic survey was completed online or on paper and the pandemic survey was completed online. The pre-pandemic survey collected demographic data. We collected risk of household FI, income and participation in food assistance programmes at both time points. The early-pandemic survey asked about participation in the school meals programme, COVID-19-related illness, and economic and daily lifestyle changes.

### Risk of household food insecurity

We administered the two-item Household Food Insecurity Screen which has been validated against USDA’s gold standard Household Food Security Survey Module (HHFSSM), showing high sensitivity and specificity among young children, adolescents and adults^(16–18)^. The screen is referred to as ‘risk of household FI’ because it captures marginal food security, which has been associated with adverse health and developmental outcomes for children, but yields higher rates of FI than the HHFSSM^(19)^. Families were considered at risk of FI if they answered ‘sometimes’ or ‘often’ to either: (1) ‘We worried whether our food would run out before we got money to buy more’; or (2) ‘The food that we bought just did not last and we did not have money to get more’. The reference period was the past 12 months and 2 months on the pre-pandemic and early-pandemic surveys, respectively.

### Family demographics

Caregivers self-reported their race and ethnicity. Due to sample size limitations, we assigned caregivers to one of three categories for analysis: non-Hispanic White, non-Hispanic Black and Other (including Hispanic, multiracial, Asian, Native American or Alaskan Native, other, and non-response). We classified families as residing in rural, suburban or urban communities using Census Bureau designations for their child’s school or childcare centre^(20)^. We calculated family income as a percent of the federal poverty level (% FPL) using 2018 and 2019 thresholds for pre-pandemic and early-pandemic surveys, respectively^(21)^. We defined three % FPL categories (≤ 185 %, > 185–300 % and > 300 %) at each time point and calculated change in percent FPL from pre-pandemic to early-pandemic. Caregivers reported their relationship to the child, and the child’s age and sex.

### COVID-19-related illness and economic hardships

In the early-pandemic survey, caregivers reported on COVID-19 symptoms or diagnosis among family members. Caregivers also reported on changes in household monthly income (no change, increased and decreased) and employment status of at least one adult (no change, hours decreased, temporary or permanent job loss) due to COVID-19.

### Safety net programmes

Caregivers reported on participation in the SNAP and Special Supplemental Nutrition Program for Women, Infants, and Children (WIC) on pre-pandemic and early-pandemic surveys. Caregivers reported on their child’s participation in the school meal programme on the early-pandemic survey. Pre-pandemic school meal participation was defined as any participation in the school breakfast or lunch programme before COVID-19 school closures. Early-pandemic school meal participation was defined as any participation in the school meal programme in the previous 2 weeks. For SNAP, WIC and school meals, we derived four categories reflecting change from pre-pandemic to early-pandemic (stopped participating, started participating, participated pre- and early-pandemic, and never participated). We also asked caregivers to report participation in the unemployment insurance programme and receipt of the CARES stimulus payment (received, expecting and not expecting).

### Statistical analysis

To focus on families most at risk of FI, we restricted the sample to families who reported they had received or expected to receive the CARES stimulus payment OR who were not expecting to receive the stimulus payment but reported an economic hardship (decreased monthly income or decreased hours or job loss due to COVID-19). We excluded families with missing FI screening data at either time point. Our analytic sample was 496 families (Fig. 2). We described characteristics of the analytic sample and used McNemar’s test to examine changes in risk of FI, % FPL, and SNAP, WIC, and school meal participation over time. We examined differences in pre-pandemic characteristics between families included in and excluded from the analytic sample.

Using Poisson regression with robust standard errors, we examined independent associations of family demographics, COVID-19-related illness and economic hardships, and safety net programmes with early-pandemic risk of FI in unadjusted and adjusted models. Multivariable models were adjusted for family demographic characteristics (race/ethnicity, locale, early-pandemic % FPL and change in % FPL). We stratified models by pre-pandemic FI risk. Cells with ten or fewer observations pre-pandemic were not estimated. We considered two-sided tests with *P* < 0·05 statistically significant. All analyses were conducted using R version 4.0.3^(22)^.

## Results

Of 496 caregivers, 43 % were from CHAMP (child aged 3–5 years at pre-pandemic recruitment) and 57 % were from WCC (children aged 6–10 years and 11–15 years at pre-pandemic recruitment) (Table 1). Half of the caregivers (51 %) identified as non-Hispanic White, 37 % as non-Hispanic Black and 13 % were classified as Other. Over half of children (56 %) attended childcare or school in suburban areas. One-quarter of families had incomes ≤ 185 % FPL; half (54 %) had incomes > 300 % FPL pre-pandemic. Median ± interquartile range follow-up time was 17 ± 8 months. The prevalence of pre-pandemic FI risk did not differ between families included and excluded from the analytic sample, though children from included families were slightly older and more likely to attend school or childcare in a rural community (see online supplementary material, Supplemental Table 1).

**Table 1:** Sample characteristics pre-pandemic and early-pandemic

	*n* 496	
	*n*	%
Pre-pandemic study		
CHAMP	213	43
WCC	283	57
Child sex, male	236	48
Child age		
3–5 years	213	43
6–10 years	140	28
11–15 years	143	29
Locale		
Rural	128	26
Suburban	280	56
Urban	88	18
Caregiver relationship to child, mother	446	90
Caregiver race/ethnicity		
Non-Hispanic White	250	51
Non-Hispanic Black or African American	181	37
Multiracial	24	5
Hispanic or Latino any race	23	5
Asian	9	2
Native American or Alaskan Native	5	1
Other race/did not respond	4	1
Number of adults in family		
1	162	33
2	275	57
≥ 3	47	10
Number of children in family		
1	100	20
2	207	42
≥ 3	184	37
Pre-pandemic risk of food insecurity*	111	22
Early-pandemic risk of food insecurity*	123	25
Pre-pandemic % federal poverty line		
≤ 185 %	121	25
> 185–300 %	99	21
> 300 %	257	54
Pre- to early-pandemic change in % federal poverty line†		
Mean	−0·76	
sd	7·02	
Pre-pandemic SNAP participation	74	15
Pre- to early-pandemic change in SNAP participation		
Never participated	363	78
Started participating	31	7
Stopped participating	10	2
Participated pre- and early-pandemic	62	13
Pre-pandemic WIC participation	40	8
Pre- to early-pandemic change in WIC participation		
Never participated	418	90
Started participating	8	2
Stopped participating	12	3
Participated pre- and early-pandemic	28	6
Pre-pandemic school meal participation‡	394	81
Pre- to early-pandemic change in school meal participation		
Never participated	117	24
Started participating	8	2
Stopped participating	277	57
Participated pre- and early-pandemic	86	18
Early-pandemic unemployment insurance	49	10
Pandemic CARES stimulus payment		
Received	434	88
Expecting	33	7
Not expecting	27	5
Early-pandemic change in household monthly income		
No change	284	58
Decreased	193	39
Increased	16	3
Early-pandemic change in employment status		
No change	295	60
Reduced hours	100	20
Temporary or permanent job loss	97	20
Family member COVID-19 diagnosis or symptoms	49	10

CHAMP, Creating Healthy Habits Among Maryland Preschoolers; WCC, Wellness Champions for Change; SNAP, Supplemental Nutrition Assistance Program; WIC, Special Supplemental Nutrition Program for Women, Infants, and Children, CARES, Coronavirus Aid, Relief, and Economic Security.

* Risk of food insecurity defined as caregiver response of ‘sometimes’ or ‘often’ to either: (1) ‘We worried whether our food would run out before we got money to buy more’; or (2) ‘The food that we bought just did not last and we did not have money to get more’ in the past 12 months pre-pandemic and past 2 months on the pandemic survey.

† Presented as mean and standard deviation.

‡ Data on school meal participation were collected as part of the early-pandemic survey. Pre-pandemic school meal participation was defined as any participation in the school breakfast or lunch programme before COVID-19 school closures. Early-pandemic school meal participation was defined as any school meal in the previous 2 weeks.

In the early phase of the pandemic, nearly 40 % of families reported decreased monthly income or change in employment status; most (88 %) had received the CARES stimulus payment. Ten per cent of households had experienced COVID-19 illness within their family (Table 1). The proportion of families participating in SNAP increased slightly (15 % to 19 %), while the proportion participating in WIC was unchanged (see online supplementary material, Supplemental Table 2). Pre-pandemic, 81 % of children had consumed school breakfast or lunch, decreasing to 26 % following early-pandemic school closures. There was a small, non-significant increase in the prevalence of families at risk of FI, from 22 % pre-pandemic to 25 %. Fourteen per cent of families who were food secure pre-pandemic were at risk of early-pandemic FI, while 61 % of families who were at risk of FI pre-pandemic were also at risk of early-pandemic FI.

### Disparities in early-pandemic FI risk

Race/ethnicity and % FPL were associated with risk of early-pandemic FI (Table 2). Among pre-pandemic food secure families, risk of early-pandemic FI was 2·1 (95 % CI 1·1, 4·0) times higher for non-Hispanic Black families and 2·6 (1·3, 5·4) times higher for Other families compared to non-Hispanic white families. Relative to families with incomes > 300 % FPL, early-pandemic FI risk was 3·2 (1·8, 5·8) times higher for families > 185–300 % FPL and 2·5 (1·3, 4·9) times higher for families ≤ 185 % FPL in adjusted models. Among families who were at risk of FI pre-pandemic, the risk of early-pandemic FI was 3·6 (1·6, 7·8) times higher for families ≤ 185 % FPL and 2·8 (1·2, 6·4) times greater for families > 185–300 % FPL compared to families > 300 % FPL.

**Table 2:** Associations of family demographics with early-pandemic risk of household food insecurity*

	Pre-pandemic food secure				Pre-pandemic risk of food insecurity			
	RR	95 % CI	aRR†	95 % CI	RR	95 % CI	aRR†	95 % CI
Family demographics								
Race/ethnicity								
Non-Hispanic White	Ref		Ref		Ref		Ref	
Non-Hispanic Black	**3·1**	**1·8, 5·4**	**2·1**	**1·1, 4**	1·1	0·7, 1·6	0·8	0·6, 1·2
Other‡	**3·3**	**1·6, 6·7**	**2·6**	**1·3, 5·4**	1·1	0·7, 1·7	0·8	0·5, 1·2
Locale								
Rural	0·5	0·3, 1	0·6	0·3, 1·4	1	0·6, 1·7	0·7	0·4, 1·3
Suburban	Ref		Ref		Ref		Ref	
Urban	1·5	0·9, 2·7	1·3	0·7, 2·3	**1·4**	**1, 1·8**	1·1	0·8, 1·5
% FPL§								
≤ 185 %	**3·6**	**1·9, 6·8**	**2·5**	**1·3, 4·9**	**3·5**	**1·6, 7·8**	**3·6**	**1·6, 7·8**
> 185–300 %	**3·9**	**2·2, 7**	**3·2**	**1·8, 5·8**	**2·8**	**1·2, 6·4**	**2·8**	**1·2, 6·4**
> 300 %	Ref		Ref		Ref		Ref	
Change in % FPL	1	1, 1·1	1	1, 1·1	1	1, 1	1	1, 1

RR, relative risk; aRR, adjusted relative risk; % FPL, percent federal poverty line.

* Associations modelled using Poisson regression with robust standard errors. Boldface indicates statistical significance (*P* < 0·05).

† Adjusted for family demographics: caregiver race/ethnicity, locale, percent federal poverty line during early-pandemic and change in federal poverty line percentage from pre- to early-pandemic.

‡ The other caregiver race/ethnicity includes Hispanic, multiracial, Asian, Native American or Alaskan Native, other race, and did not respond.

§ As the distribution in the percent federal poverty line categories did not change pre- to early-pandemic, we used the contemporary early-pandemic measure which was calculated using 2019 poverty thresholds.

### COVID-19-related illness, economic hardships and safety net programmes

Among pre-pandemic food secure families, families that experienced early-pandemic-related economic hardships, including decreased monthly income, reduced employment hours, or temporary or permanent job loss, were more than twice as likely to be at risk of early-pandemic FI than families who did not experience a hardship (adjusted relative risk (aRR) = 2·1 (1·2, 3·6) to aRR = 2·5 (1·5, 4·1)) (Table 3). COVID-19-related illness was not associated with FI risk. Among families who experienced FI risk pre-pandemic, associations of economic hardships and COVID-19-related illness with early-pandemic FI risk were null.

**Table 3:** Associations of health and economic hardships and safety net programme participation with early-pandemic risk of household food insecurity*

	Pre-pandemic food secure				Pre-pandemic risk of food insecurity			
	RR	95 % CI	aRR†	95 % CI	RR	95 % CI	aRR†	95 % CI
Health and economic hardships								
Family member diagnosed or had symptoms of COVID-19	0·9	0·3, 2·2	1	0·4, 2·8	0·8	0·5, 1·4	0·9	0·6, 1·5
Change in income in past month								
No change/increased	Ref		Ref		Ref		Ref	
Decreased	**2·9**	**1·7, 4·8**	**2·5**	**1·5, 4·1**	**1·4**	**1, 1·9**	1·3	0·9, 1·7
Change in employment in past month								
No change	Ref		Ref		Ref		Ref	
Reduced hours	**2·5**	**1·4, 4·4**	**2·1**	**1·2, 3·6**	1·3	0·9, 1·8	1·1	0·8, 1·5
Temporary or permanent job loss	**2·4**	**1·3, 4·3**	**2·3**	**1·2, 4·4**	1·3	0·9, 1·8	1	0·7, 1·4
Safety net programmes								
Unemployment insurance								
Received	1·1	0·5, 2·5	1	0·4, 2·4	1·3	1, 1·8	**1·4**	**1·1, 1·9**
Did not receive	Ref		Ref		Ref		Ref	
CARES stimulus payment								
Received‡	0·6	0·3, 1·1	**0·5**	**0·3, 0·9**	–		–	
Expecting/not expecting‡	Ref		Ref		Ref		–	
Pre- to early-pandemic change in SNAP participation								
Stopped participating‡	–		–		–		–	
Started participating	2·1	0·9, 4·8	0·8	0·2, 2·8	1·1	0·6, 2·1	0·9	0·5, 1·5
Participated pre- and early-pandemic	**3·4**	**1·8, 6·3**	2·2	0·9, 5·5	**1·7**	**1·2, 2·3**	1·2	0·9, 1·7
Never participated	Ref		Ref		Ref		Ref	
Pre- to early-pandemic change in WIC participation								
Stopped participating‡	–		–		–		–	
Started participating‡	–		–		–		–	
Participated pre- and early-pandemic	**2·5**	**1·2, 5·5**	1·4	0·6, 3·3	**1·4**	**1·1, 1·9**	1·1	0·8, 1·5
Never participated	Ref		Ref		Ref		Ref	
Pre- to early-pandemic change in school meal participation								
Stopped participating	**0·5**	**0·3, 0·9**	0·8	0·4, 1·3	0·8	0·6, 1·1	0·9	0·7, 1·2
Started participating‡	–		–		–		–	
Participated pre- and early-pandemic	**0·1**	**0, 0·4**	**0·2**	**0·1, 0·9**	**0·3**	**0·1, 0·9**	0·6	0·2, 1·4
Never participated	Ref		Ref		Ref		Ref	

RR, relative risk; aRR, adjusted relative risk; CARES, Coronavirus Aid, Relief, and Economic Security; SNAP, Supplemental Nutrition Assistance Program; WIC, Special Supplemental Nutrition Program for Women, Infants, and Children.

* Associations modelled using Poisson regression with robust standard errors. Boldface indicates statistical significance (*P* < 0·05).

† Adjusted for demographic characteristics: caregiver race/ethnicity, locale, percent federal poverty line during early-pandemic and change in federal poverty line percentage from pre- to early-pandemic.

‡ Estimates for cells with ten or fewer observations pre-pandemic are not presented.

Among pre-pandemic food secure families, receipt of the CARES stimulus payment was associated with 50 % reduced risk of early-pandemic FI (aRR = 0·5 (0·3, 0·9)). Compared to families who did not participate in the school meal programme, participation pre- and early-pandemic was associated with 80 % reduction in FI risk (aRR = 0·2 (0·1, 0·9)). Among families at risk of FI pre-pandemic, continued school meal participation pre- and early-pandemic was also protective (RR = 0·3 (0·1, 0·9)), although not significant following adjustment. Continued (pre- and early-pandemic) SNAP and WIC participation were each associated with increased early-pandemic FI risk, compared to those who never participated, in unadjusted models. Unemployment insurance was associated with risk of early-pandemic FI among families at risk pre-pandemic.

## Discussion

In a statewide sample of families with children, the prevalence of FI risk in spring/summer 2020 was unchanged from pre-pandemic levels; however, disparities by race/ethnicity and socio-economic status were observed. Furthermore, families experiencing an early-pandemic-related economic hardship were at increased risk of becoming food insecure compared to families without reported hardships. Two safety net programmes, the CARES stimulus payment and school meals, were associated with reduced risk of early-pandemic FI for families who were food secure prior to the pandemic. For families with a history of FI risk pre-pandemic, none of the safety net programmes examined mitigated early-pandemic FI risk.

In our study, families of colour and low-income families were at increased risk of experiencing FI early in the pandemic, consistent with national trends observed during the pandemic^(1)^. Nearly, 40 % of our families reported a job loss or reduction in their hours and reduced monthly income, similar to the unemployment rate (48 %) observed in a national longitudinal study of households with incomes below $75 000^(23)^. Direct payments to families may buffer against the negative effects of the pandemic. Among families who were food secure pre-pandemic, families who received the CARES stimulus payment during the initial phase of the pandemic when unemployment was at its peak were less likely to experience FI risk. Similarly, other studies have shown that the expanded unemployment insurance benefit protected adults who lost their jobs during the early-pandemic from food shortages and the expiration of the expanded benefit was associated with increased risk of food shortages and worsening mental health^(23,24)^.

Schools play an important role in feeding children, providing up to two-thirds of children’s daily nutritional needs^(25)^. Pre-pandemic food secure families who participated in the school meal programme pre-pandemic and continued to participate in the early phase of the pandemic were less likely to be at risk of FI, suggesting that school meals were an important resource for many families. Emergency authorisations and innovations to the school meals programme (e.g. delivering meals via school bus, distributing multiple meals at once) may serve as a blueprint for feeding children during future school closures^(26,27)^. However, implementation research is needed to understand barriers to accessing early-pandemic meals as over half of children in our sample stopped participating in the meals programme following school closures^(28)^. P-EBT may have offered additional protection to eligible families, but we could not assess participation as P-EBT was initiated while our survey was in the field.

Through multiple pre-pandemic studies, SNAP and WIC have been shown to reduce rates of FI and alleviate its consequences on children’s health and well-being^(29–32)^. In our study, there were few changes in the rates of SNAP and WIC participation. Given the decreases in monthly income, there may be increased need to facilitate access to these programmes. The finding that continued SNAP and WIC participation before and early in the pandemic were not associated with reducing the risk of FI suggests that the benefits were not adequate to alleviate the food shortages associated with the early-pandemic. Alternatively, the additional protections from the increased SNAP benefit (average increase $165/month) may have been cancelled out by rising food costs^(3)^. In addition, SNAP and WIC may have strengthened families’ food security without altering their FI risk. SNAP and WIC remain important safety net programmes for millions of families with children.

For families who experienced risk of FI before the pandemic, early-pandemic-related economic hardships and COVID-related illness were not associated with increased risk of FI, though families may have experienced more severe FI or hardships in other areas. Safety net programmes enhanced or created at the onset of the pandemic were intended to mitigate the negative consequences of the public health emergency; however, for families with a history of pre-pandemic FI risk, none of the safety net programmes examined were associated with reduced early-pandemic FI risk. These findings suggest the need for policymakers to consider additional support for vulnerable families with children^(33)^.

### Strengths and limitations

Although the statewide sample was diverse with respect to child age, race/ethnicity, income and locale, it was not representative of the state, limiting the generalisability of the findings. Participation in the COVID-19 survey was limited to families with internet access and may have excluded families with lower incomes, though demographic differences in families included and excluded from the analysis were minimal. We relied on caregivers’ report of receipt of or participation in safety net programmes and did not attempt to verify their responses. Additionally, small cell counts precluded estimating associations of selected categories of safety net programme participation. Strengths include longitudinal data on families before and during the pandemic and repeated use of the validated two-item Household Food Insecurity Screen, reducing recall bias and measurement error.

## Conclusions

Early-pandemic FI among families with children was associated with disparities by race/ethnicity and socio-economic status. For families at risk of FI prior to the pandemic, associations with safety net programmes were null, suggesting additional support is needed for these vulnerable families. For families who were food secure pre-pandemic, associations with safety net programmes other than the CARES stimulus payment and school meals were largely null, though they may have alleviated hardships in areas that were not measured.

The CARES stimulus payment and school meals were associated with reduced early-pandemic FI risk, and presumably protection for children’s health and development. Income support has been shown to strengthen families’ ability to care for themselves and their children, often through better nutrition and support for health-related social needs^(34,35)^. The need for income support was acute during the initial phases of the pandemic in response to rising unemployment but it is likely to be needed throughout the pandemic and other times when economic stability is threatened. Disparities in FI often persist following disasters; thus, the disruptions to children’s health and development associated with risk of FI are likely to be disproportionately borne by children in the most marginalised families^(4,5,7,8,36,37)^. Strategic policies and programmes to reduce disparities in FI risk that will persist beyond the pandemic are critical national investments to strengthen the health and well-being of all children.

## Supplementary material

For supplementary material accompanying this paper visit https://doi.org/10.1017/S136898002100481X. click here to view supplementary material
