# Supplementary material for: Pre-pandemic to early-pandemic changes in risk of household food insecurity among Maryland families with children
Source: Public Health Nutr. 2021 Dec 10;25(6):1701–10. doi: 10.1017/S136898002100481X (PMC8943228; doi:10.1017/S136898002100481X)
Supplement: Supplementary file 1 [file S136898002100481Xsup001.docx]

Pre-pandemic to early-pandemic changes in risk of household food insecurity among Maryland families with children Supplementary Material

Supplemental Table 1. Characteristics of participants included and excluded from the analysis

Supplemental Table 2. Pre- to early-pandemic changes in sample characteristics

**Supplemental Table 1. Characteristics of participants included and excluded from the analysis**

|  | **Included**  **(n = 496)** | **Excluded**  **(n = 567)** | **P** |
| --- | --- | --- | --- |
| **Pre-pandemic characteristics** | **N (%)** | **N (%)** |  |
| Household food insecurity screen |  |  | 0.42 |
| Secure | 385 (78%) | 412 (75%) |  |
| At risk of food insecurity | 111 (22%) | 135 (25%) |  |
| Baseline study |  |  | < 0.01 |
| CHAMP | 213 (43%) | 313 (55%) |  |
| WCC | 283 (57%) | 254 (45%) |  |
| Child age |  |  | < 0.01 |
| 3-5 years | 213 (43%) | 312 (55%) |  |
| 6-10 years | 140 (28%) | 137 (24%) |  |
| 11-15 years | 143 (29%) | 119 (21%) |  |
| Locale |  |  | 0.02 |
| Rural | 128 (26%) | 119 (21%) |  |
| Suburban | 280 (56%) | 312 (55%) |  |
| Urban | 88 (18%) | 136 (24%) |  |
| Caregiver relationship to child, mother | 446 (90%) | 82 (94%) | 0.28 |
| Caregiver race/ethnicity |  |  | 0.55 |
| Non-Hispanic white | 250 (51%) | 265 (47%) |  |
| Non-Hispanic Black | 181 (37%) | 222 (40%) |  |
| Other | 62 (13%) | 71 (13%) |  |
| % Federal poverty line |  |  | 0.19 |
| ≤185% | 121 (25%) | 158 (30%) |  |
| >185-300% | 99 (21%) | 93 (18%) |  |
| >300% | 257 (54%) | 274 (52%) |  |
| Number of adults in home |  |  | 0.32 |
| 1 | 162 (33%) | 203 (37%) |  |
| 2 | 275 (57%) | 283 (52%) |  |
| >3 | 47 (10%) | 57 (10%) |  |
| Number of children in home |  |  | 0.26 |
| 1 | 100 (20%) | 121 (22%) |  |
| 2 | 207 (42%) | 251 (45%) |  |
| >3 | 184 (37%) | 180 (33%) |  |

**Supplemental Table 2. Pre- to early-pandemic changes in sample characteristics**

|  | **Pre- pandemic**  **(n = 496)** | **Pandemic**  **(n = 496)** |  |  |
| --- | --- | --- | --- | --- |
|  | **N (%)** | **N (%)** | **Δ** | **p** |
| Household food insecurity screen^a^ |  |  |  | 0.27 |
| Food secure | 385 (78%) | 373 (75%) | -3% |  |
| At risk of food insecurity | 111 (22%) | 123 (25%) | +3% |  |
| % Federal poverty line |  |  |  | 0.76 |
| ≤ 185% | 121 (25%) | 120 (25%) | 0% |  |
| >185 - 300% | 99 (21%) | 104 (21%) | 0% |  |
| > 300% | 257 (54%) | 263 (54%) | 0% |  |
| Food assistance |  |  |  |  |
| SNAP | 74 (15%) | 93 (19%) | +4% | <0.01 |
| WIC | 40 (8%) | 36 (7%) | -1% | 0.50 |
| School meals^b^ | 394 (81%) | 126 (26%) | -55% | <0.01 |

Abbreviations: SNAP, Supplemental Nutrition Assistance Program; WIC, Special Supplemental Nutrition Program for Women, Infants, and Children

^a^ Risk of food insecurity define as caregiver response of “sometimes” or “often” to either: (1) “We worried whether our food would run out before we got money to buy more”; or (2) “The food that we bought just did not last and we did not have money to get more.” in the past 12 months pre-pandemic and past 2 months on the pandemic survey.

^b^ Data on school meal receipt were collected as part of the pandemic survey. Pre-pandemic school meal receipt was defined as any receipt of school breakfast or lunch before COVID-19 school closures. Pandemic school meal receipt was defined as any school meal in the previous two weeks.
